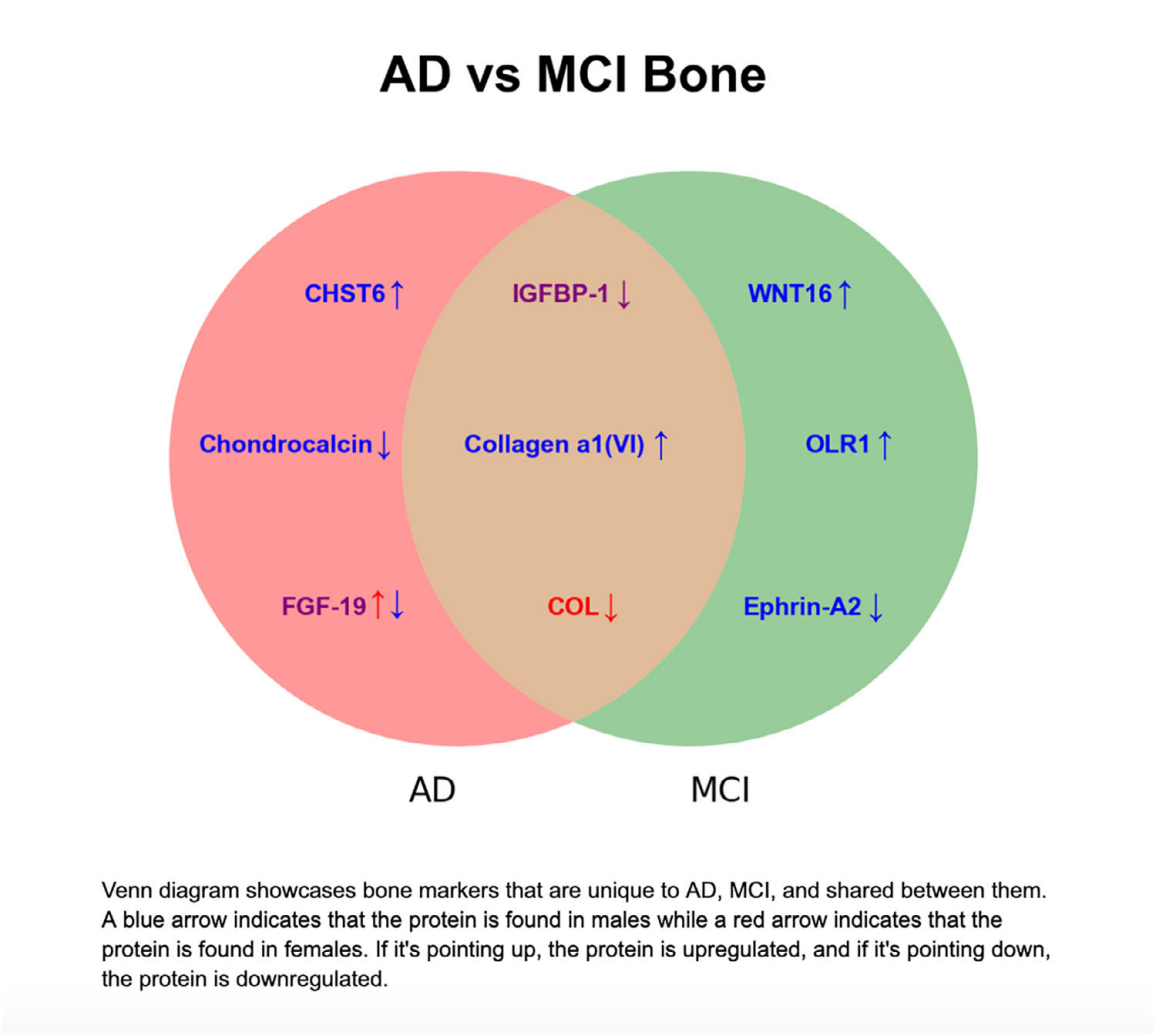# Age‐related bone associated comorbidity indicated as changes in the plasma bone and senescence markers in Alzheimer's disease patients

**DOI:** 10.1002/alz70856_104186

**Published:** 2025-12-25

**Authors:** Siddharth Kaipa, Sai R Meka, Ramesh V Nair, Divya Channappa, Hamilton S Oh, Patricia Moran‐Losada, Jarod E Rutledge, Elizabeth Mormino, Michael Zeineh, Tony Wyss‐Coray, Victor Henderson, Vidyani Suryadevara

**Affiliations:** ^1^ Stanford University, Stanford, CA, USA; ^2^ Rush University Medical Center, Chicago, IL, USA; ^3^ Stanford University School of Medicine, Stanford, CA, USA

## Abstract

**Background:**

Dementia represents a significant health and societal challenge, identified as a primary cause of mortality worldwide and closely associated with the aging population. A 24‐year follow up study indicated that socioeconomic status affects the risk of multimorbidity including dementia, frailty, and disability, with multimorbidity showing the strongest association with mortality. There is evidence that fractures pose a risk factor for dementia and individuals with dementia had a higher risk for falls and fractures. The common risk factors for dementia and impaired bone health are aging, ApoE ^7^, vitamin D and lifestyle choices. To determine if there are global changes in bone health along the progression of AD, we performed a cross‐sectional evalution of serum levels of proteins related to bone metabolism.

**Methods:**

Plasma from people wiht Alzheimer's disease (AD, n = 95), Mild cognitive impairment (MCI, n = 134), and healthy controls (HC, n = 394) was used to extract plasma proteins. 2865 proteins were identified after heparin‐bound proteins were enriched using heparin affinity chromatography and subsequently subjected to tandem mass spectrometry at Stanford ADRC, followed by filtering for bone markers.

**Results:**

In MCI, Collagen A1(VI), WNT16, and OLR1 are upregulated in males whereas IGFBP‐1, Ephrin‐A2, and IGFBP‐1 are downregulated compared to HC. In females, COL is downregulated in MCI compared to HC. On the other hand during AD, bone related proteins Collagen A1(VI) and CHST6 are upregulated in males, whereas FGF‐19, IGFBP‐1, Chondrocalcin, FGF‐19, and IGFBP‐1 are downregulated compared to HC. In females, FGF‐19 is upregulated and IGFBP‐1 and COL are downregulated in AD compared to HC. The senescent markers upregulated in MCI are WNT16 and HGFm whereas IL32 in AD compared to AD patients.

**Conclusions:**

These findings provide evidence of sex‐dependent progressive bone tissue alterations, and plasma biomarkers of senescence revealing a potential link between age associated neurodegeneration and bone health.